# Charles Bonnet Syndrome With Superimposed Delirium

**DOI:** 10.7759/cureus.27570

**Published:** 2022-08-01

**Authors:** Chelsea Karson, Christopher Kang, Brittany Albrecht, Geoffrey Levin

**Affiliations:** 1 Psychiatry and Behavioral Sciences, Advocate Lutheran General Hospital, Park Ridge, USA; 2 Psychiatry and Behavioral Sciences, Midwestern University Chicago College of Osteopathic Medicine, Downers Grove, USA

**Keywords:** charles bonnet syndrome plus, mental health stigma, deafferentation theory, perceptual release theory, antipsychotic, visual hallucination, delirium, cbs, charles bonnet syndrome

## Abstract

Charles Bonnet Syndrome (CBS) is diagnosed when a patient who is psychiatrically intact experiences visual hallucinations in the setting of significant visual acuity or field loss. The exact pathophysiology of the CBS hallucinations remains largely unknown. The main theories include the deafferentation theory and perceptual release theory. There are suspected neurotransmitters involved, including acetylcholine and dopamine. There is no defined treatment protocol with medication for CBS, but various psychotropic medications have been used with varying degrees of remission of symptoms.

This case report describes a 64-year-old male with Charles Bonnet Syndrome in the setting of superimposed delirium. We note the different medications that were trialed to reduce his CBS symptoms and decrease episodes of behavioral disturbances. Clinical features of this rare syndrome with superimposed delirium are summarized in hopes of providing directions for management and future study.

## Introduction

What is perceived by an individual but lacks evidence of external existence is a hallucination. The word "hallucination" was defined by French psychiatrist Esquirol in the early 1800s [1]. Eighteenth century naturalist Charles Bonnet described the hallucinations his grandfather experienced as his vision declined in his 1760 book *"Essai analytique sur les faculties de l'ame"* [2]. Bonnet theorized the hallucinations were from continued activity in the no longer stimulated visual processing centers of his brain. More than 200 years later, the condition is named Charles Bonnet Syndrome but remains poorly understood and in need of further study [3].

Charles Bonnet Syndrome (CBS) is diagnosed when a patient who is psychiatrically intact experiences visual hallucinations (VH) in the setting of significant visual acuity or field loss [3]. CBS is a diagnosis of exclusion. The VH experienced by patients who have CBS could be explained by other conditions such as retinal tears, retinal detachment, Lewy body dementia, brain tumors, or psychotic disorders [4]. Patients should be evaluated and diagnosed by a team composed of a psychiatrist, ophthalmologist, and a neurologist after ruling out other causes such as substance use disorders, psychotic disorders, and organic brain conditions [5].

Theories have circulated regarding the pathophysiology of the CBS hallucinations, but the exact cause remains unknown. The main theory, the deafferentation theory, postulates that sensory deafferentation results in the disinhibition of visual cortical regions, leading them to fire spontaneously. In other regions of the body, deafferentated neurons are associated with changes suggestive of denervation hypersensitivity [6]. An alternative theory, the perceptual release theory suggests that perceptual pathways are inhibited by higher cortical centers. Therefore, when perception is decreased, these pathways are no longer suppressed, resulting in hallucinations. When deprived of stimulation, serotonin levels are lowered within the visual cortex. Other neurotransmitters, specifically acetylcholine and dopamine, are also suspected to be involved [4]. 

It is not well-known what percentage of patients who have vision loss experience CBS, with estimates varying from 11-15% to 20% and even up to 40%. This is likely because of the inconsistency of the diagnostic criteria of CBS. What constitutes a VH is not well-defined and may be caused by many conditions, as described above [3, 7]. CBS symptoms include VH that can be simple or complex [3, 7, 8]. Examples of simple images include lines, light flashes, and geometric shapes. Examples of complex images include people, animals, or scenes [3, 7]. The hallucinations can be distressing in a significant number of patients with CBS [8]. CBS hallucinations occur more commonly with sensory deprivation and when the patient's eyes are open rather than closed [9]. The duration of CBS also varies, with patients reporting experiencing hallucinations for a year to five years or more [8]. Studies suggest that social isolation, poor quality relationships, and loneliness are associated with CBS [7].

Treatment of CBS should begin before it occurs. The Sydney Health Partners Emergency Department (SHaPED) trial has shown that educating at-risk patients and encouraging patients to report symptoms before they manifest resulted in better outcomes. The trial also provides a framework for how to best treat patients; it includes psychoeducation, career support, and CBT therapy [7, 10]. Evidence suggests supportive care is often the most effective treatment for CBS. Patients who have received education about CBS, understanding that the hallucinations are the result of visual impairment and are not dangerous, have better outcomes [11]. There are no official recommendations for treatment with medication, but a variety of medications, including selective serotonin reuptake inhibitors (SSRIs), antipsychotics, anti-anxiety, and anticonvulsants, have been used with varying degrees of success. Support groups and behavioral exercises have also shown some benefits to patients. Improvement in vision can resolve or lessen CBS symptoms [7, 10]. Antipsychotic medications, through dopamine antagonism, are often used to treat patients suffering from CBS. Case reports show patients have been successfully treated with olanzapine, risperidone, quetiapine, and haloperidol [5, 12,13]. 

Written below is the case of a patient who was diagnosed with CBS after a thorough neurological workup. Although he initially had insight into his VH, his condition changed. The complexity of the VH in the setting of untreated multiple sclerosis (MS) resulted in a unique case of delirium and posed challenges in treating his behavioral disturbances. 

## Case presentation

The patient, a 64-year-old male with a history of untreated multiple sclerosis (MS) and vision loss secondary to dense grade five nuclear sclerotic bilateral cataracts and major depressive disorder (MDD), was brought to the emergency department after being found wandering by emergency medical services. The ophthalmology consult found that the patient was positive for dense grade five nuclear sclerotic cataracts bilaterally and displayed a typical seeking behavioral gaze consistent with legal blindness. His VH on presentation were vivid and complex, including elaborate scenery. The patient retained insight; he reported that he was aware the VH were not real because they were clearer than his vision. The patient's family reported episodes of behavioral disturbances over the prior three months that followed a pattern of waxing and waning insight consistent with delirium. The family also raised concerns over declining cognition over several years, attributed to untreated MS.

**Figure 1:**
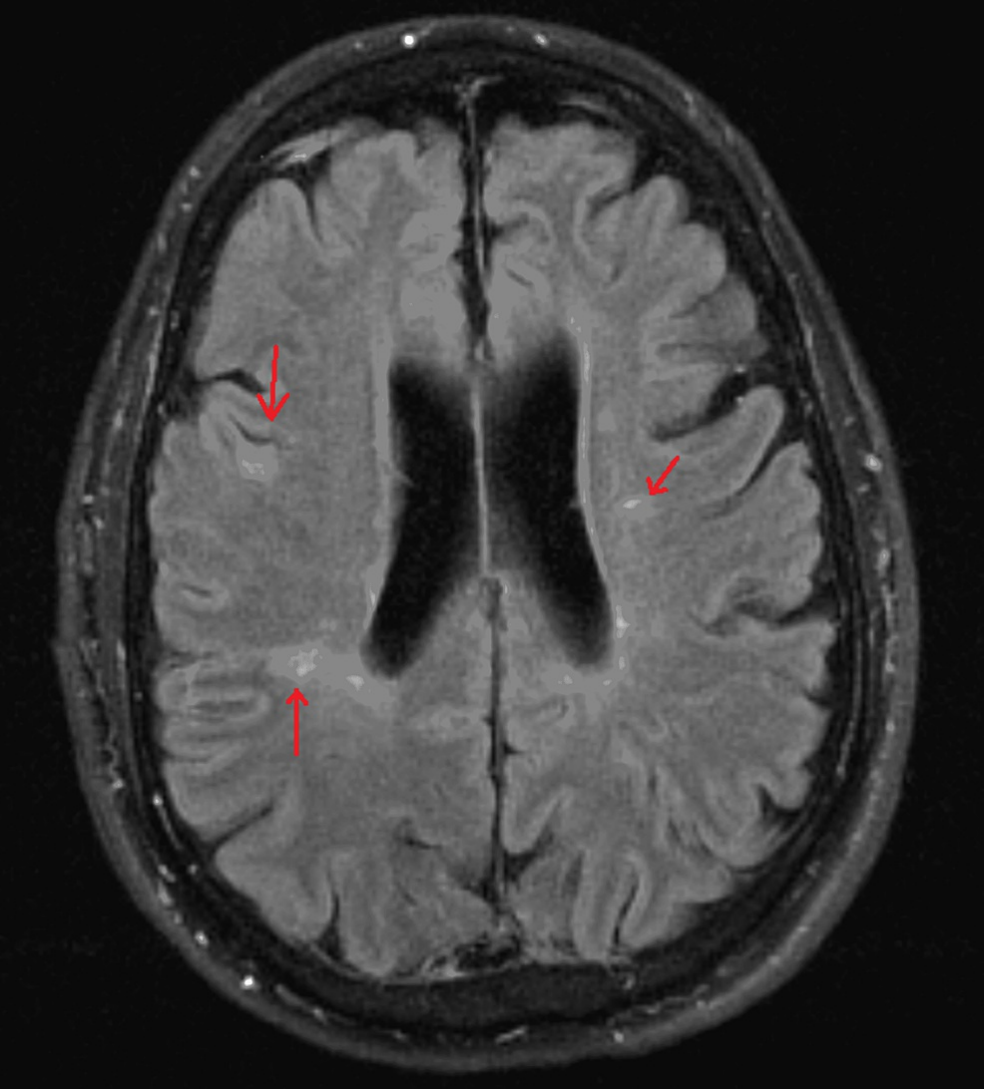
Bilateral T2-FLAIR periventricular and deep white matter signal abnormalities seen on the axial image of the brain, which given the patient's clinical history, are consistent with chronic demyelinating disease Signal abnormalities are indicated by the red arrows. FLAIR - fluid attenuated inversion recovery

**Figure 2:**
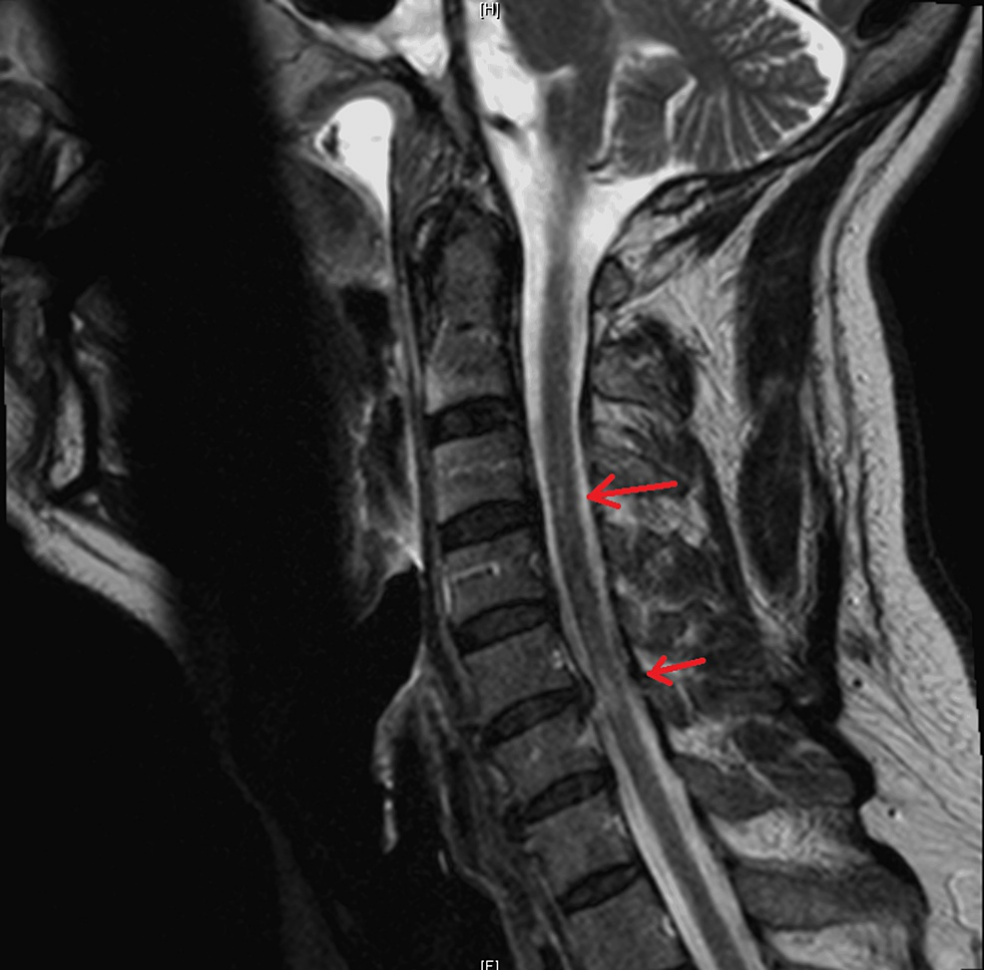
T2 hyperintense signal abnormalities seen at C3 and C6-C7, seen on sagittal cervical spine Signal abnormalities are indicated by the red arrows.

**Figure 3:**
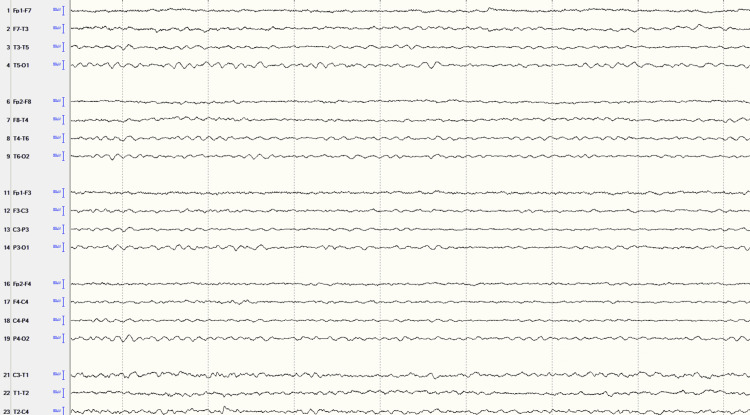
Routine EEG The EEG shows 8 HZ background and is not well organized, indicating mild diffuse encephalopathy.

The patient's MDD was treated with long-term escitalopram 10mg. He was diagnosed with MS in 1997, which was left untreated. During this hospitalization, the neurologist considered his MS too late to treat. MRI brain in 2019 (Figure 1) revealed findings of chronic supratentorial and infratentorial demyelinating disease without acute demyelinating plaque. MRI cervical spine in 2019 (Figure 2) showed chronic demyelinating disease. Routine EEG (Figure 3) revealed mild diffuse encephalopathy. He denied recent alcohol or illicit drug use. The outpatient neuropsychiatry and neurology team had started lamotrigine 25mg daily and olanzapine 2.5mg at bedtime to treat the VH.

During the first hospitalization, olanzapine was increased to 5mg three times a day and later consolidated to 15mg given each evening. Lamotrigine was discontinued as it was subtherapeutic and not indicated for CBS or delirium. He was discharged after several uneventful days. He returned seven days later after waking up to VH of three hostile men in his room and accidentally hitting his wife.

During his second hospitalization, olanzapine was increased to 20mg nightly. His delirium began to improve, but the VH remained unchanged. After four days, valproic acid 500mg twice daily was started with a modest improvement of agitation. Eight days later, the patient continued to struggle with behavioral symptoms, although valproic acid was at a therapeutic level. Haloperidol 2mg twice a day was then started with marked improvement. Haloperidol was further increased two days later to 2mg daily and 4mg at 7pm resulting in adequate control of behavioral disturbances without significant changes to his VH. The patient was then discharged to a nursing home on a final medication regimen of haloperidol 2mg daily and 4mg at 7pm, valproic acid 500mg twice daily, and olanzapine 20mg nightly for treatment of CBS with superimposed delirium and escitalopram 10mg daily for treatment of depression.

## Discussion

CBS represents an area of medicine in need of further study. VHs are often alarming and not reported by patients who fear stigma. It is likely that many patients who have CBS remain undiagnosed and untreated, contributing to the large range of reported prevalence. One 2009 study found that only 9% of CBS patients were seen by a healthcare provider because of the stigma surrounding mental health. Also concerning is a lack of knowledge of CBS in the medical community, which puts patients at risk of delayed treatment or misdiagnosis [3]. Studies show that only 9-36% of patients who sought medical attention were given information about CBS [8]. The prevalence of CBS in the United States will likely increase as life expectancy increases due to age-related vision loss. One way that healthcare providers can better serve patients is by asking patients about hallucinations, thereby ensuring CBS is not missed and providing reassurance that their experience is not atypical [3].

Research into the psychological impact of CBS on patients is insufficient. Approximately 70% of patients do not feel CBS negatively impacts their lives, but 25% report they find the hallucinations unpleasant. Another study found that 32% of patients feel their lives are negatively affected by CBS [4]. As previously stated, CBS is poorly defined, which makes diagnosis difficult. Additionally, there are multiple health conditions that can cause vision loss and development of CBS and confounding factors, which result in a greater challenge.

There is no standardized treatment or standard of care for CBS, complicating treatment and potentially making it more challenging. There has not been a double-blind, placebo-controlled study to identify the most effective treatment for CBS. Physicians treating patients who have CBS do not have sufficient data to guide their care. In some cases, successful treatment was achieved with antidepressants and anticonvulsants. Limited case reports have displayed evidence for mirtazapine, risperidone, venlafaxine, and donepezil [14]. The pharmacologic treatment which may be best for patients with CBS remains unknown [10].

Here, a patient with a complex medical history who had initially presented with VH and preserved insight subsequently became delirious, eventually responding best to olanzapine and haloperidol, with some benefit from valproic acid. In this case, the treatment approach started with olanzapine, a potent antipsychotic, with partial response. The olanzapine administration resulted in diminished episodes of confusion; however, the complex and vivid nature of his hallucinations, which he intermittently perceived as not real, remained. The consideration was made to add an anticonvulsant or add another first-generation antipsychotic. The valproate was tried first, resulting in a greater, but still partial, response. Haloperidol, then, had the best response, but consideration was given as to how, when, and if to discontinue the olanzapine. Considering the level of our patient's delirium and agitation, it was thought his breakthrough agitation would lead to the need for increasing doses of haloperidol as needed. To minimize the risk of extrapyramidal symptoms, both olanzapine and haloperidol were continued.

## Conclusions

Although many years have passed since Charles Bonnet Syndrome was first studied, there is much to learn about the condition. This case illustrates the need for further research into the pathogenesis and treatment of CBS. Had the patient received care in the community, he may have avoided hospitalization and had a smoother treatment course. Vivid hallucinations characterize CBS, but much study is required before physicians and their patients will gain a clear understanding of the condition.
